# circTAF8 Regulates Myoblast Development and Associated Carcass Traits in Chicken

**DOI:** 10.3389/fgene.2021.743757

**Published:** 2022-01-04

**Authors:** Kan Li, Weichen Huang, Zhijun Wang, Yangfeng Chen, Danfeng Cai, Qinghua Nie

**Affiliations:** ^1^ Department of Animal Genetics, Breeding and Reproduction, College of Animal Science, South China Agricultural University, Guangzhou, China; ^2^ National-Local Joint Engineering Research Center for Livestock Breeding, Guangdong Provincial Key Lab of Agro-Animal Genomics and Molecular Breeding, and Key Laboratory of Chicken Genetics, Breeding and Reproduction, Ministry of Agriculture, Guangzhou, China

**Keywords:** circTAF8, snps, flanking introns, non-coding RNA, muscle development, carcass traits, chicken

## Abstract

Recent studies have shown that circular RNAs (circRNAs) play important roles in skeletal muscle development. CircRNA biogenesis is dependent on the genetic context. Single-nucleotide polymorphisms in the introns flanking circRNAs may be intermediate-inducible factors between circRNA expression and phenotypic traits. Our previous study showed that circTAF8 is an abundantly and differentially expressed circRNA in leg muscle during chicken embryonic development. Here, we aimed to investigate circTAF8 function in muscle development and the association of the SNPs in the circTAF8 flanking introns with carcass traits. In this study, we observed that overexpression of circTAF8 could promote the proliferation of chicken primary myoblasts and inhibit their differentiation. In addition, the SNPs in the introns flanking the circTAF8 locus and those associated with chicken carcass traits were analyzed in 335 partridge chickens. A total of eight SNPs were found associated with carcass traits such as leg muscle weight, live weight, and half and full-bore weight. The association analysis results of haplotype combinations were consistent with the association analysis of a single SNP. These results suggest that circTAF8 plays a regulatory role in muscle development. These identified SNPs were found correlated with traits to muscle development and carcass muscle weight in chickens.

## Introduction

Skeletal muscle development directly impacts carcass yield for meat consumption and is affected by heredity, nutrition, breed, sex, and environment (Fortin et al., 1987; Houba et al., 2004; Halevy et al., 2006). From a genetic point of view, muscle development is under the precise regulation of a series of specific genes and signals, mainly including the myogenic regulatory factor family and myocyte enhancer factor-2 family, the paired box transcription factors Pax3 and Pax7, and myostatin (Pas and Visscher, 1994; Grefte et al., 2007). In addition to these coding genes, a growing number of studies have found that noncoding RNAs also play important roles in muscle development (Luo et al., 2013; Cai et al., 2017; Simona et al., 2018).

Circular RNAs (circRNAs) are closed circular RNA molecules formed by back-splicing of a precursor mRNA, lacking a 3ʹ end poly-A tail and an 5ʹ end cap structure (Kristensen et al., 2019). CircRNAs are widely present in eukaryotic animals and participate in various biological processes. Studies have found that circRNAs are closely related to myogenesis, the transformation of muscle fiber types, and skeletal muscle diseases (Kyei et al., 2020; Li et al., 2020; Chen et al., 2021). Our group has previously reported that circSVIL (Ouyang et al., 2018a), circHIPK3 (Chen et al., 2019), and circFGFR2 (Chen et al., 2018) promote the proliferation and differentiation of chicken myoblasts. Biological functions of circRNAs mainly include acting as miRNA sponges, interacting with various RNA-binding proteins (RBPs), and cap-independent translation themselves (Chen and Yang, 2015). Currently, studies on the mechanism whereby circRNAs regulate muscle development mainly focus on the interaction of circRNAs with miRNA (Ouyang et al., 2018a; Chen et al., 2019).

Single-nucleotide polymorphisms (SNPs) are variations of only one nucleotide variation and are widely used in studying animal and plant genetics. To date, numerous SNP markers have been identified to be associated with various important economic traits of chickens (Gorbach et al., 2010; Niknafs et al., 2014). The three SNPs of the growth hormone receptor have been found and genotyped in an F2 full-sib chicken population. G6631778A is related to body weight at various ages, dressed weight, subcutaneous fat thickness, and hatching weight in the roosters. G6631778A is only related to the 28-day-old body weight in the hens (Ouyang et al., 2008). Several SNPs of amylase alpha 1A were associated with leg muscle weight and daily gain (Zhang et al., 2021). Because of SNPs’ wide distribution, high marker density, and high genetic stability, they have become an indispensable tool in chicken genetic breeding. With the in-depth study on circRNAs, studies have shown that SNPs could affect the formation of circRNAs and change their expression level (Paraboschi et al., 2018; Liu et al., 2019; Gao et al., 2021). The multiple-sclerosis-associated SNPs on the STAT3 gene affect the expression level of circRNA has_circ_0043813 (Paraboschi et al., 2018). The rs12196996 polymorphism in the introns flanking circFOXO3 can change circFOXO3 expression and increase the risk of coronary artery disease (Zhou et al., 2020). The biogenesis of circRNAs is influenced by *cis*-acting elements and *trans*-acting splicing factors, both of which require the participation of circRNA-flanking introns (Kristensen et al., 2019). SNPs in introns flanking circRNAs may modulate back-splicing of circRNA precursors, thereby affecting the production of circRNAs.

We have previously shown that circTAF8 is one of the top ten abundantly expressed circRNAs in chicken leg muscle at three different time points in embryonic development (GSE89355) (Ouyang et al., 2018b), indicating that circTAF8 functions in muscle development. In this study, we assessed the function of circTAF8 in the proliferation and differentiation of primary myoblasts and analyzed the association of the SNPs in the introns flanking circTAF8 with chicken carcass traits. Accordingly, this study aimed to assess whether phenotypic traits are associated with circRNAs and the SNPs in the circRNA-flanking introns.

## Materials and Methods

All animal experiments and sampling procedures used in this study were strictly implemented in accordance with the regulations of the ethics committee of laboratory animals of South China Agricultural University (approval ID: SCAU#2020C030). All samples and carcass-trait data were collected in Guangzhou Kwangfeng Industrial Co., Ltd. Guangzhou Kwangfeng Industrial Co., Ltd., is the animal experimental unit operated under South China Agricultural University.

### Experimental Animal and Sample Collection

A total of 335 healthy partridge chickens of the M3 line (77 males and 258 females) were selected to screen for SNPs. Blood samples were collected in anticoagulant tubes containing 0.5 M EDTA, and E.Z.N.A.® Blood DNA Mini Kit (Omega Bio-tek, Norcross, GA, United States) was used to isolate the genomic DNA from blood. The concentration and quality of DNA were determined using NanoDrop One (Thermo Fisher Scientific, Seattle, WA, United States) and 1% agarose gel electrophoresis. The DNA samples were stored in an ultra-low temperature refrigerator at –80°C for later use.

The phenotypic traits mainly included live weight before slaughter (LWBS), carcass weight (CW), half-bore weight (HBW), full-bore weight (FBW), pectoral muscle weight (PMW), leg muscle weight (LMW), wing weight (WW), foot weight (FW), head weight (HW), heart weight (HW), liver weight (LW), stomach weight (SW), abdominal fat weight (AFW), shank length (SL), and shank diameter (SD). These obtained data were quantified and analyzed in Microsoft Excel 365 (Microsoft Corporation, Redmond, WA, USA).

In addition, four 7-week-old white Recessive Rock (WRR) chickens with similar weights were selected. The heart, liver, spleen, lung, kidney, pectoral, leg chicken, cerebellum, epencephalon, and abdominal fat were collected to characterize the tissue expression profiles of circTAF8 and TAF8.

### Genotyping SNPs *via* Polymerase Chain Reaction

Primers based on the TAF8 gene sequence provided by NCBI (accession number: NC_052,555.1) were designed using the Oligo 7 software version 7.56 (Molecular Biology Insights, Cascade, CO, USA) and synthesized by Beijing Tsingke Biotechnology Co., Ltd. (Beijing, China). The information about the primers used is shown in Supplementary Table S1. PCR was performed in a total volume of 30 μl including the following: 2 μl DNA template, 15 μl 2× Flash PCR Master Mix (CWBio, Beijing, China), 2 μl forward and reverse primers each, and 9 μl ddH_2_O. The PCR program was as follows: pre-denaturation 95°C for 5 min; 35 cycles of denaturation at 94°C for 25 s, annealing at 60°C for 25 s, and extension at 72°C for 10 s; final extension at 72°C for 5 min. The PCR products were analyzed *via* 1% agarose gel electrophoresis.

### Isolation, Culture, Differentiation, and Transfection of Chicken Primary Myoblasts

Primary myoblasts were isolated from the leg muscle of chick embryos (eggs were from Zhuhai Yuhe Agriculture and Animal Husbandry Co., Ltd.) on day 11, as previously described (Cai et al., 2017). The isolated leg muscles were washed with phosphate-buffered saline (PBS; Gibco, Carlsbad, CA, USA) containing 1% penicillin/streptomycin (Gibco, CA, USA), and visible skin and bone tissue were removed using sterile forceps. The minced muscle tissue was digested with 0.25% trypsin-EDTA (Gibco, CA, USA) at 37°C for 20 min. The cell suspension after digestion was filtered and centrifuged at 1,000 *g* for 5 min. The supernatant was discarded, and the precipitated cell pellet was suspended in the growth medium, consisting of RPMI-1640 medium (Gibco, CA, USA), 20% fetal bovine serum (Gibco, CA, USA), and 1% penicillin/streptomycin. A twice 40-min interval attachment was used to purify myoblast. Then, cells were counted and seeded in cell culture dishes and cultured in an incubator at 37°C in a 5% CO_2_-humidified atmosphere. After the cell density reached 95%–100%, the medium was replaced with the differentiation medium (RPMI 1640 Medium, 2% horse serum [Solarbio, Beijing, China], and 1% penicillin/streptomycin).

All cells were transfected using Lipofectamine 3000 reagent (Invitrogen, CA, USA) by following the manufacturer’s directions.

### RNA Extraction, cDNA Synthesis, and RT-PCR

Total RNA was extracted according to the SteadyPure Universal RNA Extraction Kit (Accurate Biology, Wuhan, China) instructions. The concentration and integrity of extracted RNA were tested for using NanoDrop One and 1% gel electrophoresis. Evo M-MLV RT Kit (Accurate Biology, Wuhan, China) was used for reverse transcription. The cDNA obtained by transcription was used for the next step of RT-PCR. The PCR amplification procedure was as follows: pre-denaturation at 95°C for 20 s; 40 cycles of denaturation at 95°C for 20 s, and annealing and extension at (58–60) °C for 30 s. The GAPDH gene was used as an internal reference gene. All relative RNA expression was calculated using 2^−ΔΔ CT^ method (Livak and Schmittgen, 2001). The primer information is shown in Supplementary Table S1.

### CircTAF8-Overexpressed Plasmid Construction

Forward and reverse primers based on the general rules of PCR-primer design were designed to amplify the linear sequence of circTAF8. After the design was completed, an EcoRI restriction site, a forward circularization-mediating sequence, and an AG acceptor were added in the 5ʹ end of the forward primer; a BamHI restriction site, a reverse circularization -mediating sequence, and a GT donor were added in the 5ʹ end of the reverse primer. The linear sequence of circTAF8 was amplified *via* PCR from the myoblast’s total cDNA, which was cloned into the pCD25-ciR vector by using BamHI and EcoRⅠ restriction sites.

The primer sequences were as follows:

Forward primer: CGG​AAT​TCT​AAT​ACT​TTC​AGA​GGT​CGG​GCA​GCA​AGC​ACA​C.

Reverse former: CGG​GAT​CCA​GTT​GTT​CTT​ACT​GGT​GTC​TTG​ATG​TAG​GTG​T.

### Nuclear and Cytoplasmic RNA Extraction

The total number of cells required for this experiment was about 2 × 10^7^. First, the cells were washed 3 times with precooled PBS; then, the cells were scraped off using a cell scraper, collected in a 50-ml centrifuge tube, and centrifuged at 1,000 *g* for 10 min. Subsequently, each cell pellet was resuspended in 1 ml cell-disruption buffer (2 M KCl, 1 M MgCl_2_, 1 M pH 7.5 Tris–HCl, 0.5 mM DTT) and then incubated on ice for 10 min. Afterward, the cells were transferred to a pre-baked Dounce cell homogenizer (Solarbio, Beijing, China) and homogenized with 15 strokes using a pestle. The homogenates were transferred to a new microtube, gently mixed with Triton X-100 (Beyotime, Shanghai, China) at a final concentration of 0.1%, and centrifuged at 1,500 g for 15 min. The supernatants, corresponding to the cytoplasmic fraction, were transferred to a new microtube. The pellets correspond to the nuclear fraction. Nuclear and cytoplasmic RNA was extracted using the TRIzol reagent (Sigma-Aldrich, San Francisco, CA, USA).

### RNase R Digestion and Actinomycin D Treatment

Total RNA (2.5 μg) was treated with 10U RNase R (Geneseed, Guangzhou, China) for 30 min at 37°C. The RNA digestion efficiency was evaluated using electrophoresis.

Primary chicken myoblasts were cultured in the presence or absence of 5 μg/ml actinomycin D (MedChemExpress, Shanghai, China) for 6 h. Then, total RNA from various treatment groups was extracted using the SteadyPure Universal RNA Extraction Kit.

### Cell Counting Kit 8 Assay

Cells at the proliferation phase were seeded in a 96-well plate. When they reached 60% confluence, they were transfected with the indicated constructs. The CCK-8 reagent (Beyotime, Shanghai, China) was used to detect cell proliferation at 12, 24, 36, and 48 h of culture. The absorbance at 450-nm wavelength was measured using a microplate reader Model 680 (Bio-Rad, Berkeley, CA, USA).

### 5-Ethynyl-2′-Deoxyuridine Assay

As previously reported (Cai et al., 2021), Edu staining was performed using Cell Light EdU Apollo 488 *In Vitro* Kit (Ruibo, Guangzhou, China). Briefly, cells were incubated in 50 μM EdU solution for 2 h and then fixed for 30 min using 4% paraformaldehyde solution. Subsequently, 0.1% Triton X-100 solution was used for cell permeabilization. Finally, the cells were incubated with Hoechst reaction solution at room temperature in the dark for 30 min. A Leica DMi8 fluorescence microscope (Leica Microsystems, Wetzlar, Germany) and ImageJ software (U.S. National Institutes of Health, Bethesda, MD, United States) (Schneider et al., 2012) were used to acquire and analyze the image, respectively.

### Flow Cytometric of the Cell Cycle

After 48 h of transfection, cells were collected and fixed in 70% ethanol at –20°C for 24 h. Then, they were centrifuged at 1,000 *g* for 5 min, and the cell pellets were resuspended in precooled PBS. Subsequently, 0.5 ml *p*I/RNase staining buffer (BD Biosciences, Franklin Lakes, NJ, USA) was added to each sample, and the sample was incubated at 37°C in the dark for 30 min. Flow cytometric analysis was performed on a BD FACSCalibur (BD Biosciences, NJ, USA), and the data were processed using FlowJo software (Treestar, Woodburn, OR, USA).

### Immunofluorescence

The cells after transfection were washed twice with PBS, fixed in 4% formaldehyde solution for 30 min, and permeated with 0.1% Triton X-100 at room temperature for 10 min. After blocking with goat serum (Boster, Wuhan, China) for 30 min, the cells were incubated overnight with MyHC antibody (1:50 dilution; DHSB, IA, USA) at 4°C. After washing 3 times with PBS, the goat anti-mouse IgM/FITC antibody (1:1,000 dilution; Bioss, Beijing, China) was added and incubated at room temperature for 1 h. Finally, the nucleus was stained with a DAPI staining solution (Beyotime, Shanghai, China). A fluorescence microscope and ImageJ software were used to acquire and analyze the image, respectively.

### Western Blot

rBriefly, cells were washed twice with precooled PBS, mixed with the RIPA buffer (Solarbio, Beijing, China) containing 1% PMSF (Solarbio, Beijing, China), and then incubated on ice for 30 min. The cell lysates were centrifuged at 12,000 *g* for 10 min. The supernatant was mixed with the 5× SDS-PAGE loading buffer and then incubated at 95°C for 5 min. The extracted proteins were separated *via* SDS-PAGE and transferred to a PVDF membrane (400 mA, 30 min). The membrane was blocked with 5% skimmed milk powder for 1 h, then incubated with a primary antibody solution overnight at 4°C. Afterward, the PVDF membrane was washed three times with TBST solution (Beyotime, Shanghai, China) for 5 min and then incubated with the secondary antibody solution at room temperature for 60 min. The protein immunoblot results were analyzed using the Odyssey Fc system (LI-COR, Lincoln, NE, USA). β-Tubulin was used as an internal reference. The relative protein levels normalized the β-tubulin protein content. The antibody information is as follows: rabbit anti-β-tubulin (1:1,000 dilution; Bioss, Beijing, China), rabbit anti-MyoD1 (1:1,000 dilution; Bioss, Beijing, China), MyHC antibody (1:1,000 dilution; DHSB, IA, USA), goat anti-rabbit IgG H&L antibody (1:3,000 dilution; Bioss, Beijing, China), and goat anti-mouse IgG H&L antibody (1:3,000 dilution; Bioss, Beijing, China).

### Statistical Analyses

The genotype frequency and gene frequency distribution of different alleles were evaluated using Microsoft Excel. The polymorphism information content (PIC) calculation of SNPs sites was based on the formula

$$\mathrm{PIC}=1-\sum_{i=1}^{n}p_{i}^{2}-\sum_{i=1}^{n-1}\sum_{j=i+1}^{n}2p_{i}^{2}p_{j}^{2}$$

Pi and Pj are the frequencies of the “ith” and “jth” alleles, respectively, and n is the number of multiple alleles. PIC >0.5 indicates high polymorphism, PIC <0.25 indicates low polymorphism, and 0.25 < PIC <0.5 indicates moderate polymorphism.

The mixed linear model in SPSS 25.0 software (IBM, Armonk, NY, USA) was used to conduct an association analysis between different combinations of genotypes and haplotypes of polymorphic sites and chicken carcass traits. The results are shown as “mean ± standard error.” The analysis model is as follows:

$$Y_{ijlm}=\mu +G_{i}+S_{j}+f_{l}+e_{ijlm}$$

*Yijlm* is the observed value, *μ* is the overall average, *Gi* is the fixed effect of the genotype, *Sj* is the fixed effect of sex, *ƒl* is the random effect of the family, and *e*_*ijlm*_ is the random error term. Bonferroni’s test was performed to control for multiple comparisons. In addition, we used online SHesis (Yong and He, 2005) and EMBOSS Needle (Madeira et al., 2019) software to analyze the linkage disequilibrium of SNP sequence complementary information, respectively.

*A priori* power analyses were performed using G*Power software version 3.1.9.7 (Heinrich-Heine-Universität Düsseldorf, Düsseldorf, Germany) (Faul et al., 2009) based on the effect size and standard deviation of a previous publication (Chen et al., 2021) or preliminary data to achieve a significance level (α) of 0.05 and a power of 0.8. In this study, three biological replicates were included in the analyses unless stated otherwise. The statistical analyses and drawings were performed using GraphPad Prism 8.0 (GraphPad Software, CA, USA). The statistical significance of the difference between the two groups was calculated using the unpaired Student’s t-test. One-way analysis of variance (ANOVA) was used to evaluate the differences among multiple groups, followed by Tukey’s multiple-comparison test. The data were shown as mean ± standard error of the mean (SEM).

## Results

### Identification of circTAF8 Molecular Characteristics

**FIGURE 1:**
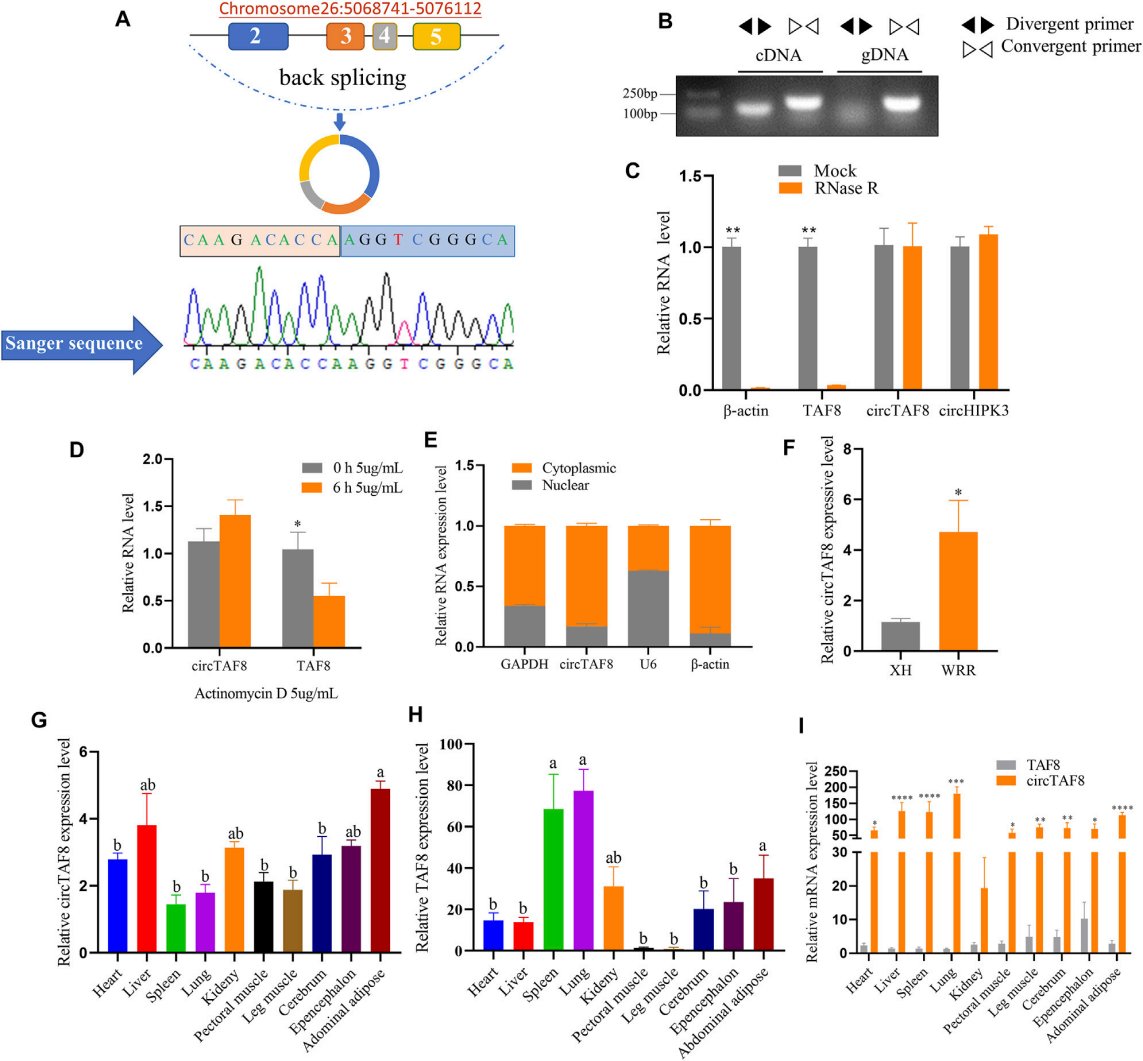
Identification of circTAF8 in chicken. **(A)** Genomic location and looping model of circTAF8 and Sanger sequencing results. **(B)** Electrophoresis results of circTAF8 amplified from cDNA and gDNA with divergent and convergent primers, respectively. **(C)** Reverse transcription-polymerase chain reaction (RT-PCR) assays to detect β-actin, TAF8, circTAF8, and circHIPK3 RNA expression levels with and without RNase R treatment. **(D)** RT-PCR to detect circTAF8 and TAF8 RNA expression levels with and without actinomycin D treatment. **(E)** RT-PCR results showing the circTAF8 subcellular localization. **(F)** RT-PCR results showing the circTAF8 expression level in XH and WRR groups. **(G)** Relative RNA expression level of circTAF8 in diverse tissues. **(H)** Relative *TAF8* RNA levels in diverse tissues. **(I)** The relative levels of *TAF8* RNA and circTAF8 in each tissue. The results of are shown as mean ± S.E.M from at least three biological replicates. The statistical significance of the differences was assessed using the unpaired Student’s *t*-test or one-way analysis of variance (ANOVA) (* *p* < 0.05; **p < 0.01; ***p < 0.001; **** *p* < 0.0001).

In this study, to verify that circTAF8 is not a technical artifact, specific divergent and convergent primers were designed based on the back-splicing sites of circTAF8. Primary myoblast cDNA and genomic DNA (gDNA) were used for PCR. The PCR products of divergent primer were sent to Beijing Tsingke Biotechnology Co., Ltd., for Sanger sequencing. The sequencing results confirmed that circTAF8 is the product of the head-to-tail cyclization of exons 2, 3, 4, and 5 of the protein-coding gene TAF8 (441 bp in total), located on chromosome 26 (Figure 1A). The electrophoresis results showed that both divergent and convergent primers could yield PCR products when cDNA was used as the template, whereas only the convergent primers could yield a product when gDNA was used as the template (Figure 1B).

CircRNAs showed resistance to digestion with exonuclease RNase R due to the lack of a 3ʹ end poly-A tail. Using RT-PCR, we quantified the expression levels of circTAF8 and its parent gene *TAF8* after RNase R digestion. Meanwhile, circHIPK3 was used as a positive control and *β-actin* as a negative control. The results showed that the expression levels of circTAF8 and circHIPK3 did not change, and linear *TAF8* and *β-actin* were almost undetectable (*p* < 0.01; Figure 1C). Then, since circRNAs usually have a longer half-life than linear RNA, we performed actinomycin D (5 μg/ml) treatment to myoblasts for 6 h for the stability evaluation of circTAF8. RT-PCR results showed no significant difference in the expression level of circTAF8 after actinomycin D treatment. In contrast, the expression of the parent gene *TAF8* was significantly reduced (*p* < 0.05; Figure 1D). Nuclear and cytoplasmic RNA extraction assays were employed to determine the subcellular localization of circTAF8. *U6*, *β-actin*, and *GAPDH* are used as controls; *U6* is mainly distributed in the nucleus, and *β-actin* and *GAPDH* are mainly distributed in the cytoplasm. The results showed that circTAF8 is present in both the cytoplasm and the nucleus (Figure 1E). Next, the expression level of circTAF8 was detected in the Xinghua (XH) and WRR chicken groups by RT-PCR, and the results showed that the expression of circTAF8 in WRR was significantly higher than in XH (*p* < 0.05; Figure 1F). XH and WRR are two breeds with different growth rates. The expression levels of circTAF8 and TAF8 in diverse tissues were quantified using qPCR. The expression profile data showed that the circTAF8 level was highest in the abdominal adipose (Figure 1G), and TAF8 was the highest in the lung (Figure 1H). Interestingly, compared with other tissues, the expressions of circTAF8 and TAF8 in muscle seems to be relatively low. In addition, the circTAF8 level in various tissues is significantly higher than that of TAF8 RNA (Figure 1I). In the lung and muscle, the circTAF8 level is approximately 200- and 50-fold the *TAF8* RNA level, respectively.

### CircTAF8 Promotes Myoblast Proliferation

**FIGURE 2:**
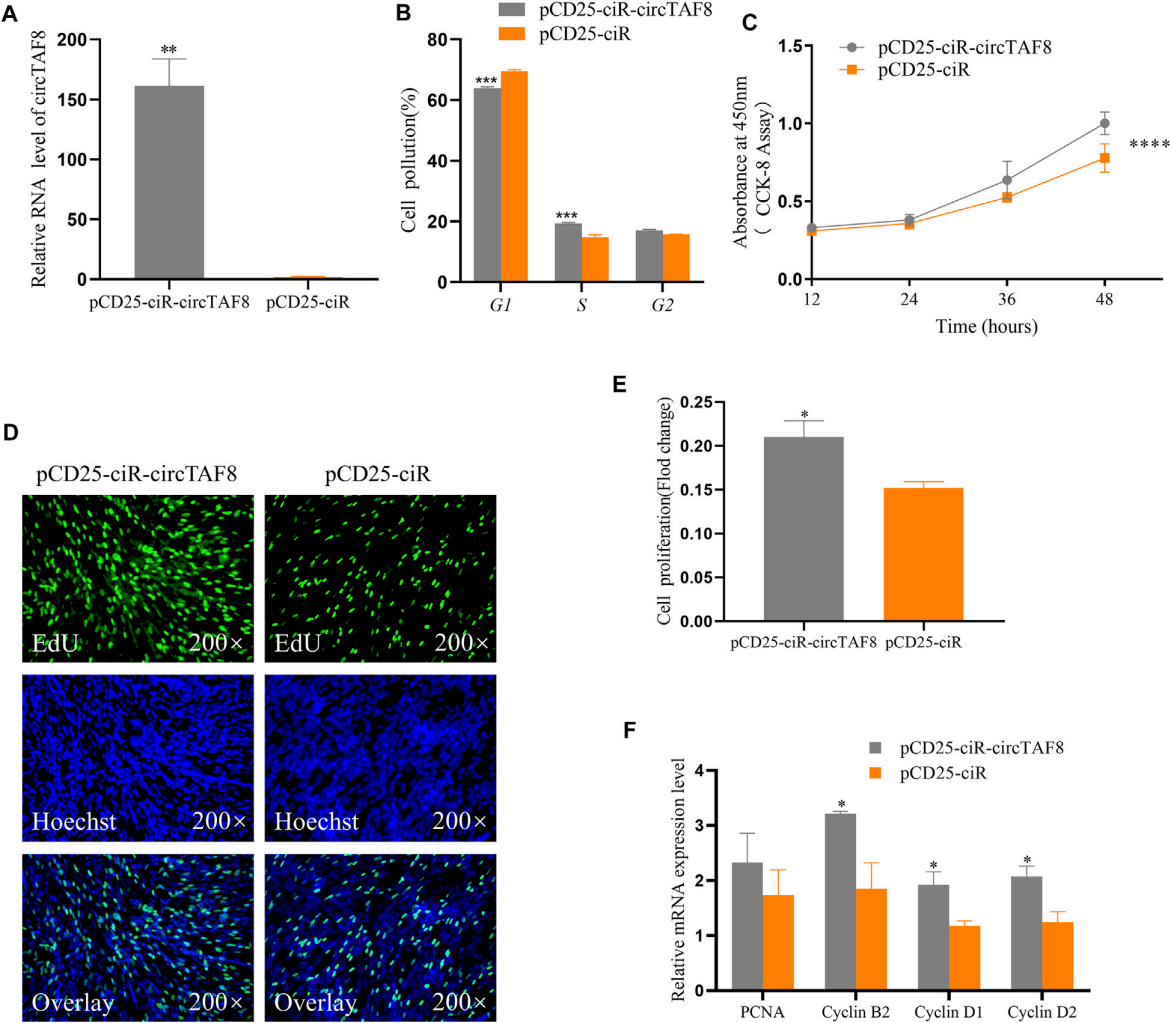
CircTAF8 promote the proliferation of myoblasts. **(A)** The relative expression level of circTAF8 after transfecting chicken primary myoblast (CPMs) with pCD25-ciR-circTAF8 or pCD25-ciR. **(B)** Cell-cycle analysis of CPMs transfected with pCD25-ciR-circTAF8 and pCD25-ciR for 36 h. **(** **C)** CCK8 analysis of CPMs transferred with pCD25-ciR-circTAF8 and pCD25-ciR. **(D)** 5-Ethynyl-2′-deoxyuridine (EdU) staining assays for CPMs transferred with pCD25-ciR-circTAF8 and pCD25-ciR for 48 h. **(E)** Fold change in proliferation rates of CPMs upon transfection with pCD25-ciR-circTAF8 or pCD25-ciR for 48 h. **(F)** Relative mRNA levels of proliferation-related genes after transfection of CPM pCD25-ciR-circTAF8 or pCD25-ciR. The data are shown as the mean ±(S.E.M) from at least three biological replicates. The statistical significance of the differences was assessed using the unpaired Student’s *t*-test (* *p* < 0.05; **p < 0.01; ***p < 0.001; **** *p* < 0.0001).

To study the effect of circTAF8 on the proliferation of primary myoblasts, primary myoblasts were transfected with the circTAF8 overexpression plasmid. The expression level of circTAF8 was detected by RT-PCR 48 h after transfection. The results showed that compared with the control group, the overexpression effect of circTAF8 reached a significant level (*p* < 0.01; Figure 2A). Then, flow cytometric analysis was performed to evaluate the cell cycle in primary myoblasts, and the results showed that the overexpression of circTAF8 increased the number of cells in the S phase and decreased the number of cells in the G1/0 phase (*p* < 0.001; Figure 2B). Besides, the CCK8 experiment was used to determine the proliferation status of myoblasts at various time points after overexpression of circTAF8. The results showed that the proliferation activity of the overexpression circTAF8 group was higher than that of the control group (*p* < 0.0001; Figure 2C). Afterward, the proliferation of the primary myoblasts was detected by EdU incorporation assays. EdU staining results showed that the ectopic expression of circTAF8 could significantly promote the proliferation of primary myoblasts (*p* < 0.05; Figures 2D,E). RT-PCR was used to detect the expression of multiple proliferation-related marker genes, and the results showed that the expression levels of Cyclin D1, Cyclin D2, and Cyclin B2 were significantly increased (*p* < 0.05; Figure 2F).

### CircTAF8 Inhibit Myoblast Differentiation

**FIGURE 3:**
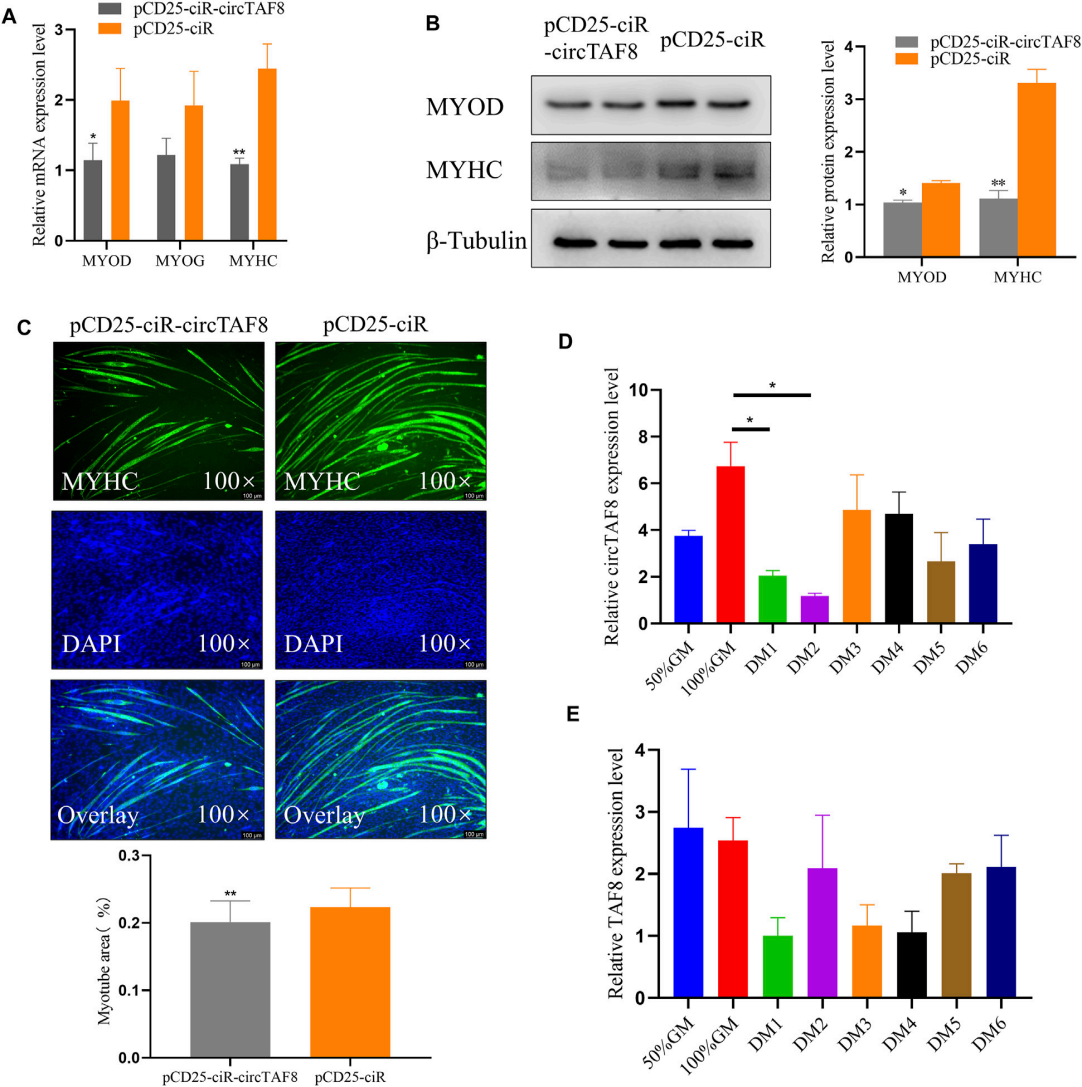
CircTAF8 inhibits myoblast differentiation. **(A)** Reverse transcription–polymerase chain reaction results showing the mRNA level of the cell differentiation-related genes in CPMs upon transfection with pCD25-ciR-circTAF8 or pCD25-ciR. **(B)** Western blot results showing the MYOD and MYOG protein levels in CPMs upon transfection with pCD25-ciR-circTAF8 or pCD25-ciR. **(C)** MyHC Immunofluorescence analysis of the myotubes transfected with pCD25-ciR-circTAF8 or pCD25-ciR. **(D)** Relative circTAF8 RNA level at various timepoints during myoblast differentiation. **(E)** Relative *TAF8* RNA levels at various timepoints during myoblast differentiation. The results are shown as mean ± S.E.M from at least three biological replicates. The statistical significance of the differences was assessed using the unpaired Student’s *t*-test or one-way analysis of variance (ANOVA) (* *p* < 0.05; **p < 0.01).

We transfected the circTAF8 overexpression plasmid into chicken myoblast cells to investigate the potential role of circTAF8 on primary myoblast differentiation. After cell density achieved 95%–100%, the growth medium was replaced by the differentiation medium. The total RNA and protein were extracted after 36 h of continuous differentiation. RT-PCR was used to detect the expression level of relevant differentiation marker genes, and the results showed that compared with the control group, the expressions of MyoD and MyHC were significantly or extremely significantly reduced (*p* < 0.05; *p* < 0.01; Figure 3A). In addition, the protein expressions of MYOD and MYHC were detected by Western blot, and the results were similar to the changes in mRNA level (*p* < 0.05; *p* < 0.01; Figure 3B). The immunofluorescence of MyHC showed that the overexpression of circTAF8 significantly reduced the myotube and the overall muscle fiber area (*p* < 0.01; Figure 3C). Afterward, we compared the expression levels of circTAF8 and TAF8 at different differentiation times of myoblasts (Figures 3D,E). The results showed that the expression level of circTAF8 during the proliferation phase was relatively high, and the expression level significantly decreased after differentiation began and then slowly increased. The expression level of TAF8 in different developmental stages of myoblasts did not reach a statistically significant difference.

### Association of the SNPs in the Introns Flanking circTAF8 With Carcass Traits

To investigate the distribution of SNPs in the introns flanking circTAF8, primers P1, P2, and P3 were used for PCR of a mixed pool of 50 DNA samples, and the PCR products were sent to Beijing Tsingke Biological Co., Ltd., for sequencing. The results show that 10 and seven SNP sites were detected in the PCR products obtained from P1 and P2, respectively, and no SNPs were detected in the PCR products of P3. The peak figures of the above sequencing results are shown in Supplementary Figure S2. All the detected SNP sites are located in the 5ʹ flanking region of the TAF8 gene. Subsequently, direct sequencing was applied to all 335 partridge chicken DNA PCR products amplified with P1 and P2, and the sequencing files were analyzed by SeqMan software (DNASTAR, Madison, WI, USA). The analysis results showed that all the above SNPs had detected three genotypes. The statistical results of the frequency of genotypes and alleles are shown in Supplementary Table S2. *Χ* ^2^ fitness test results showed that only 12 SNPs were in Hardy–Weinberg equilibrium (*p* > 0.05). The calculation results of PIC showed that except for g.-1576A > G, the remaining 16 SNPs were all moderate polymorphic, indicating that the genetic variation TAF8 was at a medium level and had great potential for selection.

The association analysis results showed that only eight SNPs were related to carcass traits (Table 1). g.-1771 G > C was significantly correlated with FBW and AFW (*p* < 0.05) and was extremely significantly correlated with SL (*p* < 0.01), and individuals of GG genotype had significantly higher FBW and SL. The locus g.-1576A > G was significantly correlated with HBW (*p* < 0.05). Both g.-1554 T > C and g.1480A > C were extremely significantly related to the LMW (*p* < 0.01), and TT and AA genotype individuals had significantly higher LMW (*p* < 0.05). The remaining four sites were associated with similar traits. g.-289 C > T, g.-288A > G, and g.-210 T > C were significantly associated with LWBS, HBW, and FBW, respectively (*p* < 0.05). g.-288A > G, g.-210 T > C, and g.-173A > G were significantly correlated with WW. g.-173A > G was also significantly related to FBW (*p* < 0.05).

**TABLE 1:** Association of eight SNPs in introns flanking circTAF8 with carcass traits.

SNP	Trait	*p*-value	Least square mean ± standard error		
g.-1771 G > C			GG (n = 6)	GC (n = 103)	CC (n = 214)
	FBW(g)	0.037	1,128.33 ± 22.70^a^	1,078.48 ± 6.30	1,071.99 ± 4.93^a^
	SL (mm)	0.004	71.88 ± 1.58^A^	66.44 ± 0.44^B^	66.82 ± 0.35^AB^
	AFW(g)	0.044	35.57 ± 4.32	26.52 ± 1.20	25.24 ± 0.95
g.-1576A > G			AA (n = 253)	AG (n = 63)	GG (n = 7)
	HBW(g)	0.030	1,290.27 ± 8.53^a^	1,252.62 ± 15.22^a^	1,333.36 ± 42.56
g.-1554 T > C			TT (n = 21)	TC (n = 105)	CC(n = 197)
	LMW(g)	0.001	253.78 ± 15.71^AB^	192.21 ± 7.55^A^	196.10 ± 5.75^B^
g.-1480A > C			AA (n = 21)	AC (n = 107)	CC(n = 195)
	LMW(g)	0.001	254.39 ± 15.71^AB^	192.36 ± 7.43^A^	196.01 ± 5.87^B^
g.-289 C > T			CC(n = 88)	CT (n = 143)	TT (n = 83)
	LWBS(g)	0.022	1,608.47 ± 9.92^a^	1,599.25 ± 7.89	1,629.83 ± 9.97^a^
	HBW(g)	0.045	1,278.47 ± 13.18	1,275.18 ± 10.16	1,312.68 ± 13.26
	FBW(g)	0.012	1,072.26 ± 6.63	1,068.22 ± 5.20^a^	1,090.64 ± 6.66^a^
g.-288A > G			AA (n = 72)	AG (n = 157)	GG (n = 85)
	LWBS(g)	0.012	1,615.45 ± 10.66	1,597.32 ± 7.77^a^	1,628.30 ± 9.84^a^
	HBW(g)	0.046	1,280.74 ± 14.32	1,274.13 ± 9.92^a^	1,311.62 ± 13.06^a^
	FBW(g)	0.007	1,076.01 ± 7.14	1,066.81 ± 5.09^A^	1,090.05 ± 6.55^A^
	WW(g)	0.034	64.23 ± 0.55	64.18 ± 0.41^a^	65.55 ± 0.5^a^
g.-210 T > C			TT (n = 67)	TC (n = 158)	CC(n = 89)
	LWBS(g)	0.017	1,612.00 ± 11.02	1,598.30 ± 7.82^a^	1,628.94 ± 9.80^a^
	HBW(g)	0.036	1,292.94 ± 14.88	1,270.77 ± 9.91^a^	1,308.98 ± 12.91^a^
	FBW(g)	0.002	1,075.76 ± 7.38	1,065.75 ± 5.11^A^	1,092.10 ± 6.50^A^
	WW(g)	0.048	64.39 ± 0.57	64.14 ± 0.41^a^	65.48 ± 0.51^a^
g.-173A > G			AA (n = 66)	AG (n = 160)	GG (n = 88)
	FBW(g)	0.004	1,075.28 ± 7.32	1,066.41 ± 5.09^A^	1,090.77 ± 6.42^A^
	WW(g)	0.047	64.53 ± 0.56	64.10 ± 0.41^a^	65.43 ± 0.50^a^

The above values are “average values ± standard errors”; in each group of SNPs, unmarked letters in the same line indicate that the difference is not significant (*p* > 0.05). When the letters are the same, lowercase letters indicate significant differences (*p* < 0.05), and uppercase letters indicate significant differences (*p* < 0.01).

### Linkage Disequilibrium and Haplotype Analysis of Eight SNPs circTAF8 Flanking Introns

**FIGURE 4:**
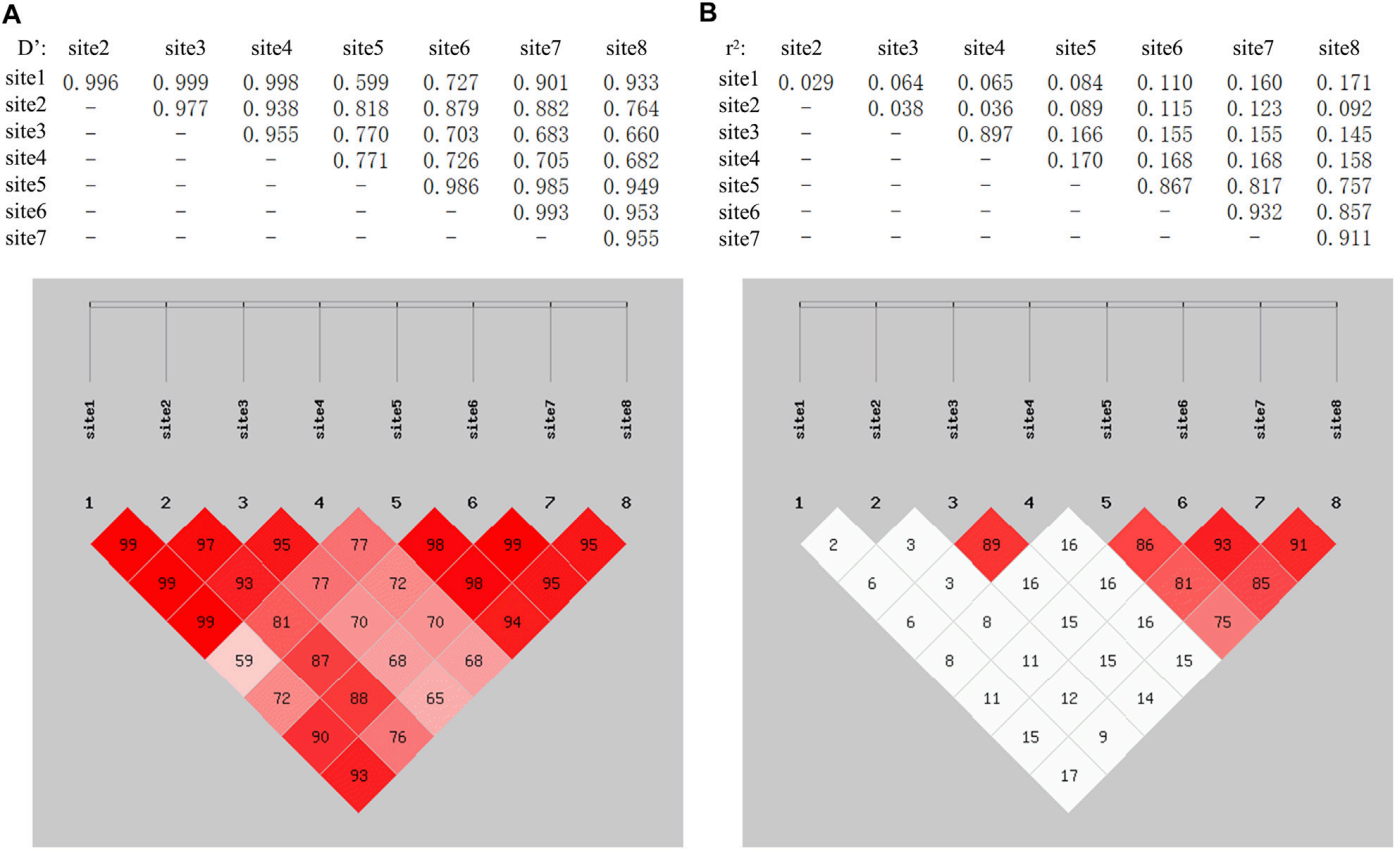
LinKage disequilibrium analysis among 8 SNPs in the introns flanking of circTAF8. The linkage among SNPs was evaluated based on Dʹ and *r* ^*2*^. **(A)** Dʹ on the left. Dʹ = 1 indicates full linkage, Dʹ = 0 indicates no linkage or linkage equilibrium. **(B)** *r* ^*2*^ on the right. *r* ^*2*^ = 1 indicates full linkage, *r* ^*2*^ > 0.33 indicates strong linkage, *r* ^*2*^ = 0 indicates no linkage or linkage equilibrium.

The SHEsis online analysis software was used to analyze the linkage disequilibrium and haplotype of the above eight SNPs. The results showed that g.-1554 T > C and g.-1480A > C can form strong linkage disequilibrium. In addition, the remaining four loci g.-289 C > T, g.-288A > G, g.-210 T > C, and g.-173A > G were in a state of strong linkage disequilibrium (Figure 4). A total of seven haplotypes were found in the g.-1554 T > C and g.-1480A > C groups. Only four haplotypes were selected for further analysis, excluding the small individuals (Table 2). These four were CCAC (6), CCCC (191), TCAC (101), and TTAA (20). Ten haplotypes consist of other four SNPs. Haplotype combinations CCAATCAA (6), CCAATTAA (65), CCAGTCAG (16), CTAGTCAG 135), and TTGGCCGG 82) were used to analyze further the association with carcass traits, except for groups with fewer individuals.

**TABLE 2:** Association of the combinations of TAF8 haplotypes with carcass traits.

Group	Traits	Least square mean ± standard error					
1		CCAC	CCCC	TCAC	TTAA	*p* value	
	LMW	196.77 ± 29.13	195.92 ± 5.93^A^	193.02 ± 7.72^B^	257.328 ± 16.15^AB^	0.024	
2		CCAATCAA	CCAATTAA	CCAGTCAG	CTAGTCAG	TTGGCCGG	*p* Value
	LWBS	1,648.41 ± 32.97	1,612.11 ± 11.12	1,582.80 ± 20.66	1,598.61 ± 8.06^a^	1,631.47 ± 10.08^a^	0.02
	HBW	1,152.30 ± 46.55^aB^	1,292.32 ± 15.19^a^	1,271.24 ± 29.05	1,276.11 ± 10.61	1,314.13 ± 13.55^B^	0.007
	FBW	1,073.51 ± 22.19	1,075.37 ± 7.48	1,057.68 ± 13.90	1,066.23 ± 5.43^A^	1,092.37 ± 6.78^A^	0.011
	LMW	198.65 ± 29.93	196.23 ± 9.68^A^	271.25 ± 18.67^ABC^	194.16 ± 6.68^B^	199.32 ± 8.86^C^	0.003

a Group 1 indicates haplotype combination of g.-1554 T > C and g.-1480A > C.

b Group 2 indicates haplotype combination of g.-289 C > T, g.-288A > G, g.-210 T > C, and g.-173A > G.

The above values are “mean values ± standard errors”; in each group of SNPs, unmarked letters in the same line indicate that the difference is not significant (*p* > 0.05). When the letters are same, lowercase letters indicate significant differences (*p* < 0.05), and uppercase letters indicate significant differences (*p* < 0.01).

The association results showed that haplotypes of g.-1554 T > C and g.-1480A > C significantly related to LWM (*p* < 0.05) and individuals of TTAA haplotypes had higher LMW (Table 2). The haplotypes composed of g.-289 C > T, g.-288A > G, g.-210 T > C, and g.-173A > G were significantly related to LWBS and FBW (*p* < 0.05) and were extremely significantly related to HBW and LMW (*p* < 0.01). Individuals of the CCAATCAA haplotype had higher LWBS, and individuals of the CCAGTCAG haplotype had higher LMW. Haplotype individuals of TTGGCCGG haplotype had higher FBW and HBW. The results of the association analysis of haplotype combinations were consistent with a single SNP.

### Complementary Pairing Analysis in Flank Sequence of circTAF8

**FIGURE 5:**
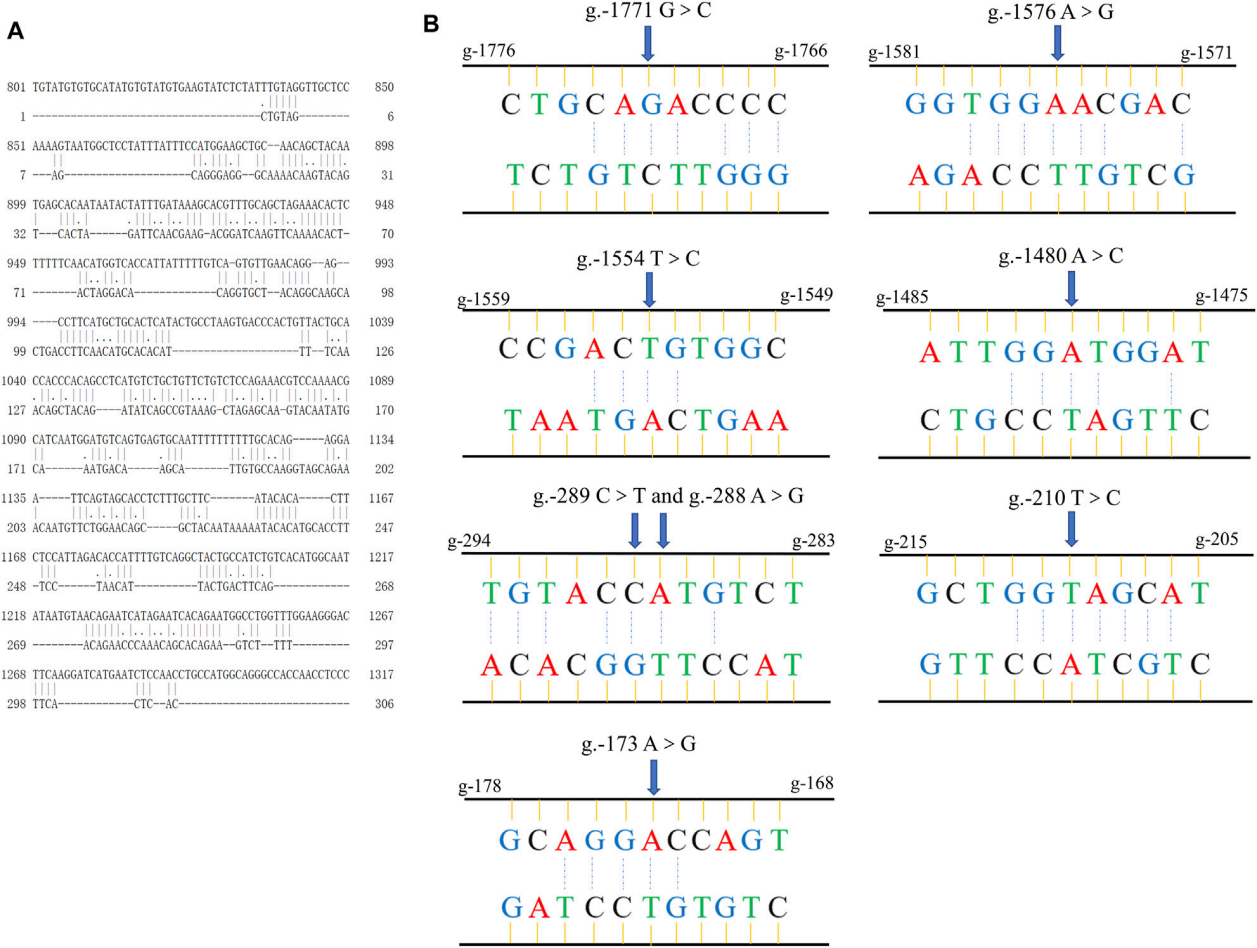
Sequence alignment information. **(A)** The global alignment of introns flanking circTAF8. **(B)** The short reverse complementary sequences of SNPs in flanking introns of circTAF8.

We first used the online software EMBOSS Needle to analyze the sequence complementarity between the flanking introns of circTAF8 (Figure 5A). The comparison results showed multiple complementary sequences in the two introns. Then, the sequences of the short fragments upstream of the eight SNP sites were aligned with the sequences of the downstream flanking introns (Figure 5B). The results showed that all SNP sites have short complementary sequences.

## Discussion

Muscle development is a complex process precisely regulated by specific genes and signaling pathways (Houba et al., 2004; Grefte et al., 2007). CircRNAs, as a new type of posttranscriptional regulator in skeletal muscle, have also been discovered in rhesus monkeys, mice, pigs, cattle, and sheep (Huang et al., 2018; Xu et al., 2018; Hao et al., 2020; Chen et al., 2021). Our previous studies have shown that circRNAs are abundantly and dynamically expressed during chicken muscle development (Ouyang et al., 2018b). According to the previous sequencing data, circTAF8 is highly and differentially expressed in the skeletal muscle at 11 embryo age, 16 embryo age, and 1-day post-hatch (Ouyang et al., 2018b), indicating that circTAF8 has a potential role in muscle development. To confirm this hypothesis, we first identified the molecular properties of circTAF8. PCR and Sanger sequencing results showed that circTAF8 was a back-splicing product derived from the two to five exons of protein-coding gene TAF8. In general, high cellular stability and longer half-life time were standard features of circRNAs due to their closed-ring structure (Kristensen et al., 2018; Kristensen et al., 2019). CircTAF8 showed resistance to digestion with exonuclease RNase R compared with the linear transcript in our results. In addition, the subcellular localization of circRNAs is closely related to their function (Wang et al., 2014). Our results showed that circTAF8 is mainly present in the cytoplasm. Multiple reports have found that most circRNAs that are related to skeletal muscle and function as miRNA sponges are located in the cytoplasm (Ouyang et al., 2018a; Chen et al., 2019; Chen et al., 2021). These results indicate that circTAF8 was reliable and stable in chicken muscle.

Breed type affects the rate of muscle development (Fortin et al., 1987). The circTAF8 expression level ranked second in circRNA sequencing data from the breast muscle of 7-week-old XH and WRR chickens (unpublished data), and the level was higher in WRR chicken than in XH chicken. XH chicken is a native slow-growing broiler; WRR is a typical fast-growing broiler (Ouyang et al., 2015). Our results presented here are consistent with sequencing data, suggesting that circTAF8 is a positive regulator of chicken muscle development. Cell confluence is essential for maintaining cell phenotype and regulating gene expression (Abo-Aziza and Zaki, 2017). Cell dynamics indicated that myoblasts would exit the exponential growth phase and begin to differentiate when they reach 100% confluence (Tanaka et al., 2011). In our study, the confluence of the chicken primary myoblasts for cell proliferation-related assays was about 60% when transfected, and the cells will fuse to 100% for 48 h after transfection. All proliferation-related assays were performed within 48 h. In comparison, the cell density for the differentiation-related study was about 70%–80% when transfected, and the myoblasts were continually differentiated using a differentiation medium for 36 h when cell density achieved 100% after transfection. Accordingly, the gain-of-function test of circTAF8 showed that circTAF8 promotes the proliferation of skeletal myoblasts and inhibits their differentiation in chickens. During myogenesis, the extent of myoblast proliferation largely determines the number of muscle fibers (te Pas, 2004). Commercial broilers usually have more muscle fibers than slow-growing chickens (Scheuermann et al., 2004; Al-Musawi et al., 2011). Therefore, the pro-proliferation effect of circTAF8 on myoblasts is essential for muscle development. However, the specific mechanism of circTAF8 in skeletal muscle regulation has not been elucidated yet, and further investigation is needed.

Genome-wide association studies have identified millions of SNPs associated with complex growth traits in chickens (Muir et al., 2008). However, how these genetic variations are related to phenotypes is often unclear. Most variation sites are located in noncoding regions such as introns and intergenic regions (Zhao et al., 2003; Abdollahi-Arpanahi et al., 2016). Interestingly, there have been reports confirming that the inverted repeat elements, complementary sequences, and certain specific motifs in the flanking introns of circRNAs can regulate the circularization of the circRNAs (Zhang et al., 2014; Ivanov et al., 2015; Conn et al., 2015). In our study, by comparing the flanking intron sequences at both ends of circTAF8, we found multiple complementary sequences between the upstream and downstream introns, indicating that the complementary pairing of introns may directly or indirectly affect the formation of circTAF8. Recent studies have reported that the polymorphism of the introns flanking circRNAs might regulate the expression of circRNAs (Burd et al., 2010; Paraboschi et al., 2018; Zhou et al., 2020). CircANRIL is transcribed from the lncANRIL gene, and the SNP within 200 bp of an ANRIL intron–exon boundary may contribute circANRIL expression, leading to risk of atherosclerotic vascular disease (Burd et al., 2010). The SNP site rs12196996 in the flanking intron of circFOXO3 is associated with the risk of coronary artery disease, due to its effect on the expression level of circFOXO3 (Zhou et al., 2020). At present, several research teams have provided databases on the correlation between circRNAs quantitative trait loci (circQTLs) and complex diseases (Ghosal et al., 2013; Liu et al., 2019; Gao et al., 2021), but research on the growth traits of animals has not been reported. As a layer of gene regulation network, the expression of circRNAs may be an intermediate phenotype that connects genetic variation and phenotypic change.

Given the importance of flanking introns in circRNA transcription level and phenotypic variation, we investigated the polymorphism of flanking introns of circTAF8. The association results showed that a total of eight SNPs were related to carcass traits, and four SNP sites (g.-289 C > T, g.-288A > G, g.-210 T > C, and g.-173A > G) are located within the 150-bp region of the 5ʹ upstream splice acceptor. These observations were consistent with previous studies, and most circQTL SNPs were close to the splicing sites (Ahmed et al., 2019; Liu et al., 2019). It is well known that skeletal muscle is the largest tissue organ in the body, accounting for about 40%–50% of the total body weight (Frontera and Ochala, 2015). Full-bore and leg muscle weight were closely related to muscle development. In this study, g.-289 C > T, g.-288A > G, g.-210 T > C, and g.-173A > G were significantly related to full-bore weight. Both g.-1480A > C and g.-1554 T > C were extremely significantly correlated with leg muscle weight. Haplotypes can usually provide more information than a single SNP site can because animal phenotypes can be affected by multiple mutations (Liu et al., 2008). The association of haplotype combinations was consistent with the results of the association analysis of a single SNP. It is worth noting that four SNPs close to the splice receptor are strongly linked to form a haplotype. This haplotype was significantly related to live weight, full-bore weight, half-bore, and leg muscle weight. In addition, we found that all eight SNP sites have short complementary sequences, indicating that the polymorphisms of these SNP sites may regulate the production of circTAF8. Based on the above results, TAF8 may serve a new SNP maker in chicken genetics and breeding, and circTAF8 may contribute to understanding the potential regulatory mechanisms of this genetic trait.

Interestingly, the results of tissue expression profiling showed that the expression level of circTAF8 was significantly higher than that of the linear transcript in various tissues of chicken; circTAF8 seems to be the major transcript of pre-TAF8. Generally, the level of circRNAs is lower than those of corresponding parent genes (Guo et al., 2014). However, some circRNAs are expressed at high levels in some unique cell lines or tissues independently of their host genes (Salzman et al., 2013; Rybak-Wolf et al., 2015). TAF8 is a TBP-binding protein of the multi-subunit transcription factor TFIID. TFIID plays a crucial role in the binding of RNA polymerase Ⅱ to transcription factors and core promoters (Trowitzsch et al., 2015). Research related to TAF8 mainly focuses on the TFIID assembly; relatively few studies focus on its function and polymorphism. Previous reports have shown that TAF8 can promote the differentiation of 3T3-L1 preadipocyte cells into adipocytes. However, it does not appear to play a role in the myogenesis of C2C12 cells (Guermah et al., 2003). Interestingly, our results showed that the expression level of the *TAF8* gene was the lowest in breast muscle and leg muscle. In addition, we found that the protein sequence of TAF8 was conserved entirely between chicken and mouse by the NCBI protein blast tool. CircRNAs are often closely related to the expression or function of their linear host genes. Various circRNAs and their host genes, such as circRBFOX2, circSVIL, circLMO7, circFGFR4, circFGFR2, and circTTN, all play a regulatory role in muscle development (Wei et al., 2017; Li et al., 2018a; Ouyang et al., 2018a; Ouyang et al., 2018b; Chen et al., 2018; Wang X. et al., 2019). However, it is worth noting that some parent genes of circRNAs related to skeletal muscle development have not been reported to be involved in muscle development, such as circTMTC1, circZNF609, circFUT10, and circHIPK3 (Li et al., 2018b; Wang Y. et al., 2019; Chen et al., 2019; Shen et al., 2019). These results suggested that pre-TAF8 is involved in regulating muscle development which might mainly be through this circular transcript. Although the function of TAF8 on chicken muscle development has not been explored, considering that circTAF8 is the main transcript, we speculate that the association effect of the flanking introns of circTAF8 with carcass traits might be mainly realized through the expression of circTAF8 in chicken. Whether circRNAs are a regulator between phenotypic traits and SNP needs to be confirmed *via* additional research.

## Conclusion

In summary, circTAF8 regulates skeletal muscle development by promoting myoblast proliferation and inhibiting myoblast differentiation. The SNPs in introns flanking circTAF8 are significantly correlated with multiple carcass traits, such as live weight, full and half-bore weight, and leg muscle weight. The association between SNPs and phenotypic traits may be achieved through the expression of circTAF8.

## Data Availability

The datasets presented in this study can be found in online repositories. The names of the repository/repositories and accession number(s) can be found in the article/Supplementary Material.

## References

[B1] Abdollahi-Arpanahi R. Morota G. Valente B. D. Kranis A. Rosa G. J. M. Gianola D. (2016). Differential Contribution of Genomic Regions to Marked Genetic Variation and Prediction of Quantitative Traits in Broiler Chickens. Genet. Sel Evol. 48 (1), 1–13. 10.1186/s12711-016-0187-z 26842494 PMC4739338

[B2] Abo-Aziza F. A. M. A A Z. (2017). The Impact of confluence on Bone Marrow Mesenchymal Stem (BMMSC) Proliferation and Osteogenic Differentiation. Int. J. Hematol. Oncol. Stem Cell Res 11 (2), 121–132. 10.1002/bem.20550 28875007 PMC5575725

[B3] Ahmed I. Karedath T. Al-Dasim F. M. Malek J. A. (2019). Identification of Human Genetic Variants Controlling Circular RNA Expression. RNA 25 (12), 1765–1778. 10.1261/rna.071654.119 31519742 PMC6859849

[B4] Al-Musawi S. L. Lock F. Simbi B. H. Bayol S. A. M. Stickland N. C. (2011). Muscle Specific Differences in the Regulation of Myogenic Differentiation in Chickens Genetically Selected for Divergent Growth Rates. Differentiation 82 (3), 127–135. 10.1016/j.diff.2011.05.012 21723031 PMC3181402

[B5] Burd C. E. Jeck W. R. Liu Y. Sanoff H. K. Wang Z. Sharpless N. E. (2010). Expression of Linear and Novel Circular Forms of an INK4/ARF-Associated Non-coding RNA Correlates with Atherosclerosis Risk. Plos Genet. 6 (12), e1001233. 10.1371/journal.pgen.1001233 21151960 PMC2996334

[B6] Cai B. Li Z. Ma M. Wang Z. Han P. Abdalla B. A. (2017). LncRNA-Six1 Encodes a Micropeptide to Activate Six1 in Cis and Is Involved in Cell Proliferation and Muscle Growth. Front. Physiol. 8, 230. 10.3389/fphys.2017.00230 28473774 PMC5397475

[B7] Cai B. Li Z. Ma M. Zhang J. Kong S. Abdalla B. A. (2021). Long Noncoding RNA SMUL Suppresses SMURF2 Production-Mediated Muscle Atrophy via Nonsense-Mediated mRNA Decay. Mol. Ther. - Nucleic Acids 23, 512–526. 10.1016/j.omtn.2020.12.003 33510940 PMC7807096

[B8] Chen B. Yu J. Guo L. Byers M. Wang Z. Chen X. (2019). Circular RNA circHIPK3 Promotes the Proliferation and Differentiation of Chicken Myoblast Cells by Sponging miR-30a-3p. Cells 8 (2), 177. 10.3390/cells8020177 PMC6406597 30791438

[B9] Chen L.-L. Yang L. (2015). Regulation of circRNA Biogenesis. Rna Biol. 12 (4), 381–388. 10.1080/15476286.2015.1020271 25746834 PMC4615371

[B10] Chen M. Wei X. Song M. Jiang R. Huang K. Deng Y. (2021). Circular RNA circMYBPC1 Promotes Skeletal Muscle Differentiation by Targeting MyHC. Mol. Ther. - Nucleic Acids 24, 352–368. 10.1016/j.omtn.2021.03.004 33868781 PMC8027698

[B11] Chen X. Ouyang H. Wang Z. Chen B. Nie Q. (2018). A Novel Circular RNA Generated by FGFR2 Gene Promotes Myoblast Proliferation and Differentiation by Sponging miR-133a-5p and miR-29b-1-5p. Cells 7 (11), 199. 10.3390/cells7110199 PMC6262629 30404220

[B12] Conn S. J. Pillman K. A. Toubia J. Conn V. M. Salmanidis M. Phillips C. A. (2015). The RNA Binding Protein Quaking Regulates Formation of circRNAs. Cell 160 (6), 1125–1134. 10.1016/j.cell.2015.02.014 25768908

[B13] Faul F. Erdfelder E. Buchner A. Lang A.-G. (2009). Statistical Power Analyses Using G*Power 3.1: Tests for Correlation and Regression Analyses. Behav. Res. Methods 41 (4), 1149–1160. 10.3758/BRM.41.4.1149 19897823

[B14] Fortin A. Wood J. D. Whelehan O. P. (1987). Breed and Sex Effects on the Development, Distribution of Muscle, Fat and Bone, and the Partition of Fat in Pigs. J. Agric. Sci. 108 (1), 141–153. 10.1017/S0021859600064212

[B15] Frontera W. R. Ochala J. (2015). Skeletal Muscle: a Brief Review of Structure and Function. Calcif Tissue Int. 96 (3), 183–195. 10.1007/s00223-014-9915-y 25294644

[B16] Gao Y. Li X. Shang S. Guo S. Wang P. Sun D. (2021). LincSNP 3.0: an Updated Database for Linking Functional Variants to Human Long Non-coding RNAs, Circular RNAs and Their Regulatory Elements. Nucleic Acids Res. 49 (D1), D1244–D1250. 10.1093/nar/gkaa1037 33219661 PMC7778942

[B17] Ghosal S. Das S. Sen R. Basak P. Chakrabarti J. (2013). Circ2Traits: a Comprehensive Database for Circular RNA Potentially Associated with Disease and Traits. Front. Genet. 4, 283. 10.3389/fgene.2013.00283 24339831 PMC3857533

[B18] Gorbach D. M. Fan B. Onteru S. K. Zhao X. Du Z.-Q. Garrick D. J. (2010). Genome-wide Association Studies for Important Economic Traits in Domestic Animals Using High Density SNP Genotyping. Iowa State. Univ. Anim. Industry Rep. 7 (1). 10.31274/ans_air-180814-980

[B19] Greco S. Cardinali B. Falcone G. F. Martelli F. (2018). Circular RNAs in Muscle Function and Disease. Ijms 19, 3454. 10.3390/ijms19113454 PMC6274904 30400273

[B20] Grefte S. Kuijpers-Jagtman A. M. Torensma R. Von den Hoff J. W. (2007). Skeletal Muscle Development and Regeneration. Stem Cell Dev. 16 (5), 857–868. 10.1089/scd.2007.0058 17999606

[B21] Guermah M. Ge K. Chiang C.-M. Roeder R. G. (2003). The TBN Protein, Which Is Essential for Early Embryonic Mouse Development, Is an Inducible TAFII Implicated in Adipogenesis. Mol. Cell 12 (4), 991–1001. 10.1016/S1097-2765(03)00396-4 14580349

[B22] Guo J. U. Agarwal V. Guo H. Bartel D. P. (2014). Expanded Identification and Characterization of Mammalian Circular RNAs. Genome Biol. 15 (7), 1–14. 10.1186/s13059-014-0409-z PMC4165365 25070500

[B23] Halevy O. Yahav S. Rozenboim I. (2006). Enhancement of Meat Production by Environmental Manipulations in Embryo and Young Broilers. World's Poult. Sci. J. 62 (03), 485–497. 10.1017/S0043933906001103

[B24] Hao Z. Zhou H. Hickford J. G. H. Gong H. Wang J. Hu J. (2020). Identification and Characterization of Circular RNA in Lactating Mammary Glands from Two Breeds of Sheep with Different Milk Production Profiles Using RNA-Seq. Genomics 112 (3), 2186–2193. 10.1016/j.ygeno.2019.12.014 31866420

[B25] Houba P. H. J. Pas M. F. W. T. Pas M. (2004). “The Muscle Regulatory Factors Gene Family in Relation to Meat Production,” in Muscle Development of Livestock Animals – Physiology, Genetics, and Meat Quality. Editors te Pas M. F. W. Everts M. E. Haagsman H. P. (Oxfordshire, UK: CABI Publishing), 201–223. 10.1079/9780851998114.0201

[B26] Huang M. Shen Y. Mao H. Chen L. Chen J. Guo X. (2018). Circular RNA Expression Profiles in the Porcine Liver of Two Distinct Phenotype Pig Breeds. Asian-australas J. Anim. Sci. 31 (6), 812–819. 10.5713/ajas.17.0651 29268579 PMC5933978

[B27] Ivanov A. Memczak S. Wyler E. Torti F. Porath H. T. Orejuela M. R. (2015). Analysis of Intron Sequences Reveals Hallmarks of Circular RNA Biogenesis in Animals. Cell Rep. 10 (2), 170–177. 10.1016/j.celrep.2014.12.019 25558066

[B28] Kristensen L. S. Andersen M. S. Stagsted L. V. W. Ebbesen K. K. Hansen T. B. Kjems J. (2019). The Biogenesis, Biology and Characterization of Circular RNAs. Nat. Rev. Genet. 20 (11), 675–691. 10.1038/s41576-019-0158-7 31395983

[B29] Kristensen L. S. Hansen T. B. Venø M. T. Kjems J. (2018). Circular RNAs in Cancer: Opportunities and Challenges in the Field. Oncogene 37 (5), 555–565. 10.1038/onc.2017.361 28991235 PMC5799710

[B30] Kyei B. Li L. Yang L. Zhan S. Zhang H. (2020). CDR1as/miRNAs-related Regulatory Mechanisms in Muscle Development and Diseases. Gene 730, 144315. 10.1016/j.gene.2019.144315 31904497

[B31] Li B. Yin D. Li P. Zhang Z. Zhang X. Li H. (2020). Profiling and Functional Analysis of Circular RNAs in Porcine Fast and Slow Muscles. Front Cell Dev Biol 8, 322. 10.3389/fcell.2020.00322/ 32528948 PMC7264268

[B32] Li H. Wei X. Yang J. Dong D. Hao D. Huang Y. (2018a). circFGFR4 Promotes Differentiation of Myoblasts via Binding miR-107 to Relieve its Inhibition of Wnt3a. Mol. Ther. - Nucleic Acids 11, 272–283. 10.1016/j.omtn.2018.02.012 29858062 PMC5992882

[B33] Li H. Yang J. Wei X. Song C. Dong D. Huang Y. (2018b). CircFUT10 Reduces Proliferation and Facilitates Differentiation of Myoblasts by Sponging miR-133a. J. Cell Physiol 233 (6), 4643–4651. 10.1002/jcp.26230 29044517

[B34] Liu N. Zhang K. Zhao H. (2008). Haplotype‐Association Analysis. Adv. Genet. 60, 335–405. 10.1016/S0065-2660(07)00414-2 18358327

[B35] Liu Z. Ran Y. Tao C. Li S. Chen J. Yang E. (2019). Detection of Circular RNA Expression and Related Quantitative Trait Loci in the Human Dorsolateral Prefrontal Cortex. Genome Biol. 20 (1), 1–16. 10.1186/s13059-019-1701-8 31109370 PMC6528256

[B36] Livak K. J. Schmittgen T. D. (2001). Analysis of Relative Gene Expression Data Using Real-Time Quantitative PCR and the 2−ΔΔCT Method. Methods 25 (4), 402–408. 10.1006/meth.2001.1262 11846609

[B37] Luo W. Nie Q. Zhang X. (2013). MicroRNAs Involved in Skeletal Muscle Differentiation. J. Genet. Genomics 40 (3), 107–116. 10.1016/j.jgg.2013.02.002 23522383

[B38] Madeira F. Park Y. m. Lee J. Buso N. Gur T. Madhusoodanan N. (2019). The EMBL-EBI Search and Sequence Analysis Tools APIs in 2019. Nucleic Acids Res. 47 (W1), W636–W641. 10.1093/nar/gkz268 30976793 PMC6602479

[B39] Muir W. M. Wong G. K.-S. Zhang Y. Wang J. Groenen M. A. M. Crooijmans R. P. M. A. (2008). Genome-wide Assessment of Worldwide Chicken SNP Genetic Diversity Indicates Significant Absence of Rare Alleles in Commercial Breeds. Proc. Natl. Acad. Sci. 105 (45), 17312–17317. 10.1073/pnas.0806569105 18981413 PMC2582254

[B40] Niknafs S. Javaremi A. N. Sadeghi M. (2014). Single Nucleotide Polymorphisms in BMPR-IB and STAT5B Genes and Their Association with Growth and Reproductive Traits in Chicken. Songklanakarin J. Sci. Tech. 36 (2), 137–142.

[B41] Ouyang H. Chen X. Li W. Li Z. Nie Q. Zhang X. (2018a). Circular RNA circSVIL Promotes Myoblast Proliferation and Differentiation by Sponging miR-203 in Chicken. Front. Genet. 9, 172. 10.3389/fgene.2018.00172 29868120 PMC5964199

[B42] Ouyang H. Chen X. Wang Z. Yu J. Jia X. Li Z. (2018b). Circular RNAs Are Abundant and Dynamically Expressed during Embryonic Muscle Development in Chickens. Dna Res. 25 (1), 71–86. 10.1093/dnares/dsx039 29036326 PMC5824844

[B43] Ouyang H. He X. Li G. Xu H. Jia X. Nie Q. (2015). Deep Sequencing Analysis of miRNA Expression in Breast Muscle of Fast-Growing and Slow-Growing Broilers. Ijms 16 (7), 16242–16262. 10.3390/ijms160716242 26193261 PMC4519947

[B44] Ouyang J. H. Xie L. Nie Q. Luo C. Liang Y. Zeng H. (2008). Single Nucleotide Polymorphism (SNP) at theGHRgene and its Associations with Chicken Growth and Fat Deposition Traits. Br. Poult. Sci. 49 (2), 87–95. 10.1080/00071660801938817 18409081

[B45] Paraboschi E. M. Cardamone G. Soldà G. Duga S. Asselta R. (2018). Interpreting Non-coding Genetic Variation in Multiple Sclerosis Genome-wide Associated Regions. Front. Genet. 9, 647. 10.3389/fgene.2018.00647 30619471 PMC6304422

[B46] Pas M. F. W. Visscher A. H. (1994). Genetic Regulation of Meat Production by Embryonic Muscle Formation - a Review. J. Anim. Breed. Genet. 111 (1-6), 404–412. 10.1111/j.1439-0388.1994.tb00477.x 21395789

[B47] Rybak-Wolf A. Stottmeister C. Glažar P. Jens M. Pino N. Giusti S. (2015). Circular RNAs in the Mammalian Brain Are Highly Abundant, Conserved, and Dynamically Expressed. Mol. Cell 58 (5), 870–885. 10.1016/j.molcel.2015.03.027 25921068

[B48] Salzman J. Chen R. E. Olsen M. N. Wang P. L. Brown P. O. (2013). Cell-type Specific Features of Circular RNA Expression. Plos Genet. 9 (9), e1003777. 10.1371/journal.pgen.1003777 24039610 PMC3764148

[B49] Scheuermann G. N. Bilgili S. F. Tuzun S. Mulvaney D. R. (2004). Comparison of Chicken Genotypes: Myofiber Number in Pectoralis Muscle and Myostatin Ontogeny. Poult. Sci. 83 (8), 1404–1412. 10.1093/ps/83.8.1404 15339017

[B50] Schneider C. A. Rasband W. S. Eliceiri K. W. (2012). NIH Image to ImageJ: 25 Years of Image Analysis. Nat. Methods 9 (7), 671–675. 10.1038/nmeth.2089 22930834 PMC5554542

[B51] Shen X. Liu Z. Cao X. He H. Han S. Chen Y. (2019). Circular RNA Profiling Identified an Abundant Circular RNA circTMTC1 that Inhibits Chicken Skeletal Muscle Satellite Cell Differentiation by Sponging miR-128-3p. Int. J. Biol. Sci. 15 (10), 2265–2281. 10.7150/ijbs.36412 31592238 PMC6775300

[B52] Tanaka K. Sato K. Yoshida T. Fukuda T. Hanamura K. Kojima N. (2011). Evidence for Cell Density Affecting C2C12 Myogenesis: Possible Regulation of Myogenesis by Cell-Cell Communication. Muscle Nerve 44 (6), 968–977. 10.1002/mus.22224 22102468

[B53] te Pas M. F. (2004). Muscle Development of Livestock Animals Physiology Genetics and Meat Quality. Oxfordshire, UK: CABI Publishing.

[B54] Trowitzsch S. Viola C. Scheer E. Conic S. Chavant V. Fournier M. (2015). Cytoplasmic TAF2-TAF8-TAF10 Complex Provides Evidence for Nuclear Holo-TFIID Assembly from Preformed Submodules. Nat. Commun. 6 (1), 1–14. 10.1038/ncomms7011 PMC4309443 25586196

[B55] Wang P. L. Bao Y. Yee M.-C. Barrett S. P. Hogan G. J. Olsen M. N. (2014). Circular RNA Is Expressed across the Eukaryotic Tree of Life. Plos One 9 (3), e90859. 10.1371/journal.pone.0090859 24609083 PMC3946582

[B56] Wang X. Cao X. Dong D. Shen X. Cheng J. Jiang R. (2019a). Circular RNA TTN Acts as a miR-432 Sponge to Facilitate Proliferation and Differentiation of Myoblasts via the IGF2/PI3K/AKT Signaling Pathway. Mol. Ther. - Nucleic Acids 18, 966–980. 10.1016/j.omtn.2019.10.019 31770673 PMC6881651

[B57] Wang Y. Li M. Wang Y. Liu J. Zhang M. Fang X. (2019b). A Zfp609 Circular RNA Regulates Myoblast Differentiation by Sponging miR-194-5p. Int. J. Biol. Macromolecules 121, 1308–1313. 10.1016/j.ijbiomac.2018.09.039 30201567

[B58] Wei X. Li H. Yang J. Hao D. Dong D. Huang Y. (2017). Circular RNA Profiling Reveals an Abundant circLMO7 that Regulates Myoblasts Differentiation and Survival by Sponging miR-378a-3p. Cell Death Dis 8 (10), e3153. 10.1038/cddis.2017.541 29072698 PMC5680912

[B59] Xu K. Chen D. Wang Z. Ma J. Zhou J. Chen N. (2018). Annotation and Functional Clustering of circRNA Expression in Rhesus Macaque Brain during Aging. Cell Discov 4 (1), 1–18. 10.1038/s41421-018-0050-1 30245844 PMC6141548

[B60] Yong Y. He L. (2005). SHEsis, a Powerful Software Platform for Analyses of Linkage Disequilibrium, Haplotype Construction, and Genetic Association at Polymorphism Loci. Cell Res 15 (2), 97–98. 10.1038/sj.cr.7290272 15740637

[B61] Zhang X.-O. Wang H.-B. Zhang Y. Lu X. Chen L.-L. Yang L. (2014). Complementary Sequence-Mediated Exon Circularization. Cell 159 (1), 134–147. 10.1016/j.cell.2014.09.001 25242744

[B62] Zhang Z. Zhong H. Lin S. Liang L. Ye S. Xu Z. (2021). Polymorphisms of AMY1A Gene and Their Association with Growth, Carcass Traits and Feed Intake Efficiency in Chickens. Genomics 113 (2), 583–594. 10.1016/j.ygeno.2020.10.041 33485951

[B63] Zhao Z. Fu Y.-X. Hewett-Emmett D. Boerwinkle E. (2003). Investigating Single Nucleotide Polymorphism (SNP) Density in the Human Genome and its Implications for Molecular Evolution. Gene 312, 207–213. 10.1016/S0378-1119(03)00670-X 12909357

[B64] Zhou Y.-L. Wu W.-P. Cheng J. Liang L.-L. Cen J.-M. Chen C. (2020). CircFOXO3 Rs12196996, a Polymorphism at the Gene Flanking Intron, Is Associated with circFOXO3 Levels and the Risk of Coronary Artery Disease. Aging 12 (13), 13076–13089. 10.18632/aging.103398 32614786 PMC7377899

