# Supplementary material for: circTAF8 Regulates Myoblast Development and Associated Carcass Traits in Chicken
Source: Front Genet. 2022 Jan 4;12:743757. doi: 10.3389/fgene.2021.743757 (PMC8764441; doi:10.3389/fgene.2021.743757)
Supplement: Supplementary file 1 [file DataSheet1.docx]

Supplementary Material

CircTAF8 full sequence

AGGTCGGGCAGCAAGCACACGACCACGCCGGCAGATAACTACTACCTGGCCCGAAGGAGGACTCTGCAGGTGGTGGTCAGCTCCTTGCTCACTGAAGCTGGTTTTGAGAGCGCTGAAAAGGCTGCGGTGGAAACACTGACAGAGATGTTACAGAGCTTATTTCTGAAATTGGGAGGAGTGCAAAGTCCTACTGTGAACATACAGCCAGAACACAGCCTACACTCTCTGATATAGTGGTTACTTTAGTGGAAATGGGTTCAATGTTGAAACGCTTCCTGCATATGCAAAGCGCTCCCAGAGGATGGTCATCACTGCCCTCCTGTGACAAATCAGCCTGTGACTCCCAAGGCCCTGACAGCTGGACAGAACAAGCCACATCCATCCCACATCCCTGGCTATTTTCCCGAGTTTCCAGATCCTCACACCTACATCAAGACACCA

Supplement Table S1. All primers information

| Primer | Primer sequence | Annealing temperature | Product size |
| --- | --- | --- | --- |
| CircTAF8 Divergent primer | F: CCCACATCCCTGGCTATTT  R: CTGCAGAGTCCTCCTTCGG | 58 | 128 |
| CircTAF8 Convergent  primer  CircTAF8 qPCR primer | F: CGGTGGAAACACTGACAGAGAT  R: GGGAGTCACAGGCTGATTTGT  F: GACAAATCAGCCTGTGACTCCC  R: ATCTCTGTCAGTGTTTCCACCG | 60  58 | 221  264 |
| TAF8 | F: ACTCTTGCAAAGAGGTCGGG  R: TCTCTGTCAGCGTTTCCACC | 60 | 157 |
| β-actin | F: AATGGCTCCGGTATGTGCAA  R: GGCCCATACCAACCATCACA | 60 | 113 |
| CircHIPK3 | F: GTTTAATCCACGCTGACCTCA  R: GACTTGTGAGGCCATACCTATA | 60 | 130 |
| PCNA | F: GTGCTGGGACCTGGGTT  R: CGTATCCGCATTGTCTTCT | 60 | 217 |
| Cyclin B2 | F: CAGTAAAGGCTACGAAAG  R: ACATCCATAGGGACAGG | 60 | 133 |
| Cyclin D1 | F: CAGAAGTGCGAAGAGGAAGT  R: CTGATGGAGTTGTCGGTGTA | 60 | 188 |
| Cyclin D2 | F: AACTTGCTCTACGACGACC  R: TTCACAGACCTCCAACATC | 60 | 150 |
| MYHC | F: CTCCTCACGCTTTGGTAA  R: TGATAGTCGTATGGGTTGGT | 58 | 213 |
| MYOG | F: CGGAGGCTGAAGAAGGTGAA  R: CGGTCCTCTGCCTGGTCAT | 58 | 320 |
| MYOD | F: GCTACTACACGGAATCACCAAAT  R: CTGGGCTCCACTGTCACTCA | 58 | 200 |
| GAPDH | F: TCCTCCACCTTTGATGCG  R: GTGCCTGGCTCACTCCTT | 60 | 146 |
| U6 | F: CGTGGGGTACAGCAGCTC  R: CAGCTCCAGGAACTCCTCCT | 60 | 253 |
| P1 | F: GGAGATTCGGCTGAAGGTTCG | 58 | 946 |
|  | R: GCCATTACTTTTGGAGCAACCTA |  |  |
| P2 | F: ATCTCTATTTGTAGGTTGCTCCA | 58 | 979 |
|  | R: CCAGCTTCAGTGAGCAAGGA |  |  |
| P3 | F: TCGTGGATGTGCTCTGTCACTA  R: GCCTCAGAAGGCTGAACACAA | 58 | 815 |
|  |  |  |  |

Supplement Table S2. The genotype and allele frequencies of 17 SNPs of circTAF8 flanking introns

| Polymorphism Site | Sample size | Genetype | Frequency | Allel | Allel frequency | *X* ^2^（p） | PIC |
| --- | --- | --- | --- | --- | --- | --- | --- |
| g.-1840 G＞A | 323 | GG(46)  GA(76)  AA(201) | 0.14  0.24  0.62 | G  A | 0.26  0.74 | 48.78(2.55×10^^-11^) | 0.25 |
| g.-1771 G＞C | 323 | GG(6)  GC(103)  CC(214) | 0.02  0.32  0.66 | G  C | 0.18  0.82 | 2.59(0.27) | 0.25 |
| g.-1614 G＞A | 323 | GG(21)  GA(48)  AA(254) | 0.07  0.14  0.79 | G  A | 0.14  0.86 | 46.73(7.15×10^^-11^) | 0.21 |
| g.-1576 A＞G | 323 | AA(253)  AG(63)  GG(7) | 0.78  0.20  0.02 | A  G | 0.88  0.12 | 1.63(0.44) | 0.18 |
| g.-1567 G＞A | 323 | GG(217)  GA(99)  AA(7) | 0.67  0.31  0.02 | G  A | 0.83  0.17 | 1.23(0.54) | 0.25 |
| g.-1554 T＞C | 323 | TT(21)  TC(105)  CC(197) | 0.07  0.33  0.60 | T  C | 0.23  0.77 | 1.83(0.40) | 0.29 |
| g.-1504T＞C | 323 | TT(62)  TC(139)  CC(121) | 0.19  0.43  0.38 | T  C | 0.59  0.41 | 3.66(0.16) | 0.37 |
| g.-1480 A＞C | 323 | AA(21)  AC(107)  CC(195) | 0.07  0.33  0.60 | A  C | 0.23  0.77 | 1.43(0.48) | 0.29 |
| g.-1472 C＞A | 323 | CC(55)  CA(84)  AA(184) | 0.17  0.26  0.57 | C  A | 0.30  0.70 | 46.9(6.45×10^^-11^) | 0.33 |
| g.-1455 T＞C | 323 | TT(56)  TC(154)  CC(113) | 0.17  0.48  0.35 | T  C | 0.41  0.59 | 0.08(0.96) | 0.37 |
| g.-945 A＞G | 294 | AA(112)  AG(52)  GG(130) | 0.38  0.18  0.44 | A  G | 0.47  0.53 | 122.28(2.8×10^-27) | 0.37 |
| g.-808 C＞A | 307 | CC(121)  CA(56)  AA(130) | 0.39  0.18  0.42 | C  A | 0.49  0.51 | 123.73(1.35×10^-27) | 0.37 |
| g.-595 C＞A | 312 | CC(144)  CA(125)  AA(43) | 0.46  0.40  0.14 | C  A | 0.66  0.44 | 3.43(0.18) | 0.35 |
| g.-289 C＞T | 314 | CC(88)  CT(143)  TT(83) | 0.28  0.46  0.26 | C  T | 0.51  0.49 | 2.48(0.29) | 0.37 |
| g.-288 C＞T | 314 | AA(72)  AG(157)  GG(85) | 0.28  0.46  0.26 | A  G | 0.48  0.52 | 0.0009(0.99) | 0.37 |
| g.-210 T＞C | 314 | TT(67)  TC(158)  CC(89) | 0.21  0.50  0.28 | T  C | 0.46  0.54 | 0.04(0.98) | 0.37 |
| g.-173 A＞G | 314 | AA(n=66) AG(n=160) GG(n=88) | 0.21  0.51  0.28 | A  G | 0.46  0.54 | 0.18(0.91) | 0.37 |

**Supplementary Figure S1**

**
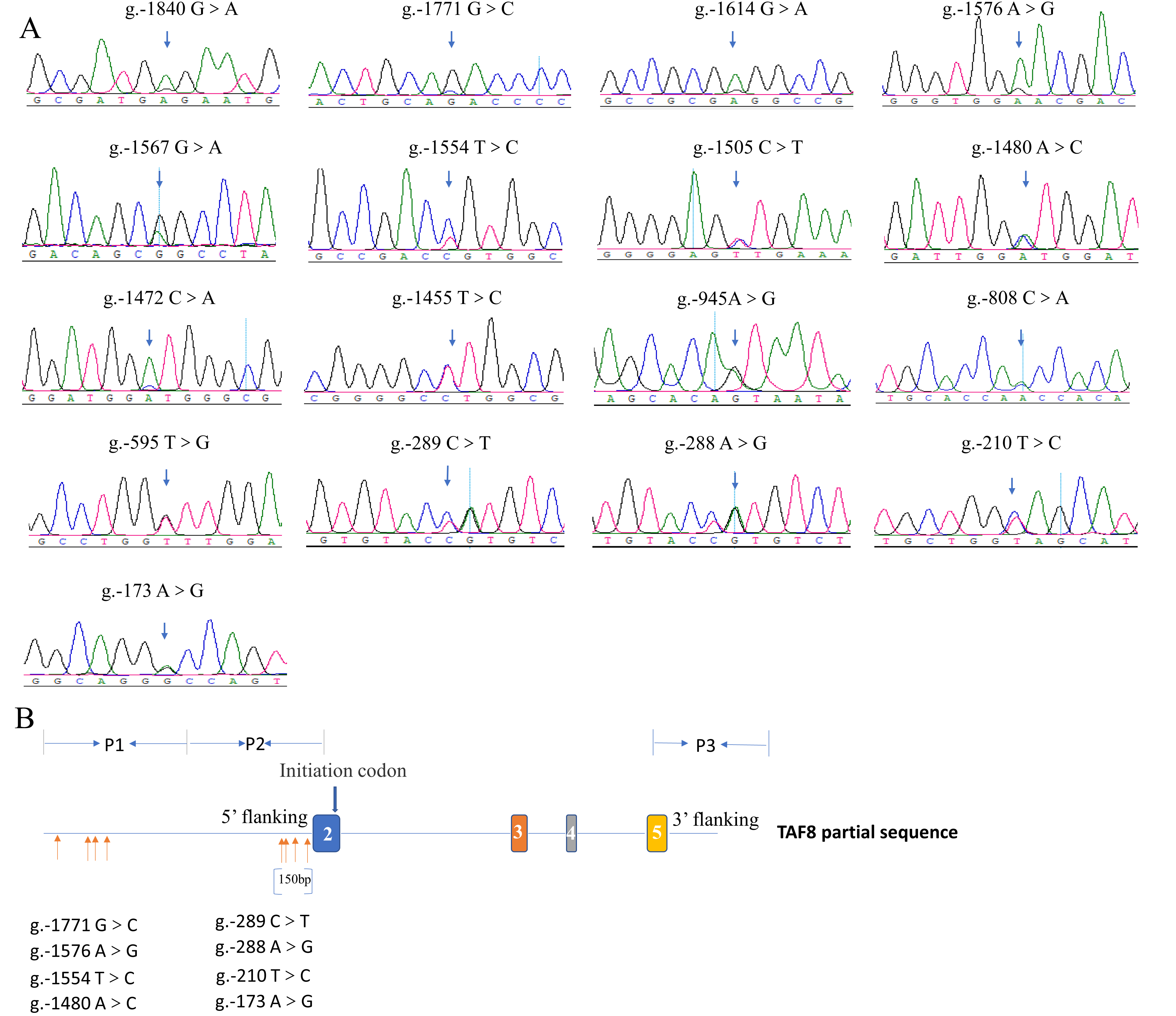
**

**Supplementary Figure 1.** The peak figure of all SNPs and distribution diagram.
